# Correlates of preschool children’s objectively measured physical activity and sedentary behavior: a cross-sectional analysis of the SPLASHY study

**DOI:** 10.1186/s12966-016-0456-9

**Published:** 2017-01-05

**Authors:** Einat A. Schmutz, Claudia S. Leeger-Aschmann, Thomas Radtke, Stefanie Muff, Tanja H. Kakebeeke, Annina E. Zysset, Nadine Messerli-Bürgy, Kerstin Stülb, Amar Arhab, Andrea H. Meyer, Simone Munsch, Jardena J. Puder, Oskar G. Jenni, Susi Kriemler

**Affiliations:** 1 Epidemiology, Biostatistics and Prevention Institute, University of Zurich, Hirschengraben 84, 8001 Zurich, Switzerland; 2 Department of Evolutionary Biology and Environmental Studies, University of Zurich, Winterthurerstrasse 190, 8057 Zurich, Switzerland; 3 Child Development Center, University Children’s Hospital Zurich, Steinwiesstrasse 75, 8032 Zurich, Switzerland; 4 Department of Clinical Psychology and Psychotherapy, University of Fribourg, Clinical Psychology and Psychotherapy, Rue PA de Faucigny 2, 1700 Fribourg, Switzerland; 5 Endocrinology, Diabetes & Metabolism Service, Centre Hospitalier Universitaire Vaudois (CHUV), Avenue Pierre Decker 2, 1011 Lausanne, Switzerland; 6 Department of Psychology, University of Basel, Missionsstrasse 62A, 4055 Basel, Switzerland; 7 Division of Pediatric Endocrinology, Diabetology and Obesity, Centre Hospitalier Universitaire Vaudois (CHUV), Rue du Bugnon 46, 1011 Lausanne, Switzerland; 8 Children’s Research Center, University Children’s Hospital Zurich, Steinwiesstrasse 75, 8032 Zurich, Switzerland

**Keywords:** Children, Preschool, Physical activity, Sedentary behavior, Correlates, SPLASHY

## Abstract

**Background:**

Identifying ways to promote physical activity and decrease sedentary time during childhood is a key public health issue. Research on the putative influences on preschool children’s physical activity (PA) and sedentary behavior (SB) is limited and has yielded inconsistent results. Our aim was to identify correlates of PA and SB in preschool children.

**Methods:**

Cross-sectional data were drawn from the Swiss Preschoolers’ Health Study (SPLASHY), a Swiss population-based cohort study. Of 476 two to six year old children, 394 (54% boys) had valid PA data assessed by accelerometry. Information on exposure data was directly measured or extracted from parental questionnaires. Multilevel linear regression modeling was used to separately assess associations between 35 potential correlates and total PA (TPA), moderate-to-vigorous PA (MVPA) and SB.

**Results:**

In total, 12 correlates from different domains were identified. TPA and MVPA were greater in boys than girls, increased with age and were positively associated with gross motor skills. Children from single parent families had a higher level of TPA and spent less time sedentary than those living with two parents. Time spent outdoors was positively associated with TPA and negatively with SB. The child’s activity temperament was related all three outcomes, whereas parental sports club membership, living area per person and neighborhood safety were associated with SB only. Fixed and random factors in the final models accounted for 28%, 32% and 22% of the total variance in TPA, MVPA and SB, respectively. Variance decomposition revealed that age, sex and activity temperament were the most influential correlates of both, TPA and MVPA, whereas the child’s activity temperament, time outdoors and neighborhood safety were identified as the most important correlates of SB.

**Conclusions:**

A multidimensional set of correlates of young children’s activity behavior has been identified. Personal factors had the greatest influence on PA, whereas environmental-level factors had the greatest influence on SB. Moreover, we identified a number of previously unreported, potentially modifiable correlates of young children’s PA and SB. These factors could serve to define target groups or become valuable targets for change in future interventions.

**Trial registration:**

Current Controlled Trials ISRCTN41045021 (date of registration: 21.03.14).

**Electronic supplementary material:**

The online version of this article (doi:10.1186/s12966-016-0456-9) contains supplementary material, which is available to authorized users.

## Background

Health behaviors, such as engaging in physical activity (PA) or sedentary behavior (SB), are formed during the foundational early years of life and track into older childhood, adolescence and adulthood [1]. Early childhood is the period when children are highly receptive to instruction and encouragement and parents may be more receptive to information regarding parenting [2]. A variety of mechanisms, including encouragement, beliefs and attitudes towards PA, role modeling, involvement and facilitation (e.g., transport and fee paying), can help to shape important attitudes and behaviors associated with PA [3]. In addition, developing healthy habits at this early stage increases the chance that they will carry over into later life.

Low levels of PA and excessive sedentary time during early childhood are associated with short- and long-term psychological and physiological health consequences [4, 5]. Supporting the development of healthy levels of participation in these behaviors during early childhood has been shown to have health, developmental, and academic benefits over time [6, 7]. It is recommended that young children engage in least two or three hours of PA at any intensity level every day and limit time spent being sedentary, particularly time spent on screen-based entertainment [8–10]. Despite the numerous documented benefits of participation in recommended levels of PA and SB, adherence to these recommendations is poor [11, 12]. Hence, identifying factors that may support or constrain PA/SB in this age group and possible at-risk demographic groups that could be targeted in future intervention studies is of paramount importance.

Young children’s PA is undertaken in a number of contexts and does not usually occur as a planned, structured activity. Research on the putative influences on young children’s PA and SB is relatively new and has yielded inconsistent results. Whereas school and peers may play an important role in older children, preschoolers are likely to experience different influences on their activity behavior, which poses challenges to transfer findings to younger age groups. Since PA and sedentary pursuits are complex behaviors influenced by factors from multiple domains that operate at a variety of ecological levels [13], the socio-ecological model of health behavior provides a theoretical framework through which potential correlates may be conceptualized. These may include demographic and biological characteristics, psychological, cognitive and emotional traits, behavioral characteristics, social and cultural variables and environmental factors. However, most studies on correlates in young children have primarily investigated a narrow range of influences and various potentially important correlates, such as the presence of siblings, single parent household status, the child’s self-regulation capacity, temperament and cognitive functioning, parent’s lifestyle behaviors, home play space and equipment, owning a dogs and neighborhood safety, remain under-researched [14]. Only a few consistent correlates of PA have been reported; these include gender, parents’ participation in child PA, parental PA, time spent outdoors and gross motor skills [14–16]. For SB, there is insufficient evidence to draw conclusions about correlates [17]. A recent systematic review of correlates and determinants in the early years concludes that gaps in the research need to be addressed by exploring potential correlates across the whole spectrum of the ecologic model within one study [14].

To gain insight into the impact of the broader contexts in which different exposures exist, we performed an in-depth evaluation of a wide range of potential correlates of PA and SB that were selected on the basis of previous research and theory primarily guided by the socio-ecological model. Using this model as a basis, our work enhances the current evidence base relating to young children’s PA behavior by investigating novel and under-researched correlates from different ecological domains that will both further our understanding of the influences and their relative importance and provide context and support for future interventions.

## Methods

### Study population

Data for the cross-sectional analysis presented in this paper were drawn from the baseline assessment of the Swiss Preschoolers’ Health Study (SPLASHY), which is a multi-site prospective cohort study including 476 children aged two to six years within two sociocultural areas of Switzerland (German and French speaking part). A detailed description of the study design has been published previously [18]. Children were recruited from 84 childcare centers in five cantons of Switzerland (Aargau, Bern, Fribourg, Vaud, and Zurich). Taken together, these cantons made up 50% of the Swiss population in 2013. Recruitment lasted from November 2013 until October 2014. Ethical approval was obtained from all local ethical committees (No 338/13 for the Ethical Committee of the Canton of Vaud as the main ethical committee) and is in accordance with the Declaration of Helsinki. Children provided oral consent and parents provided written informed consent.

### Data collection

Data collection was conducted in parallel at all study sites (Zurich/Aargau, Fribourg/Bern and Lausanne) according to standardized procedures. A multi-method approach was used including parent- and child-based self-report, observational data assessment, and physical and psychophysiological measurements at childcare centers and at home. For each childcare, the assessment included three afternoons over a period of three weeks.

### Outcome variables: physical activity and sedentary behavior

PA and SB were objectively monitored using an accelerometer (wGT3X-BT, ActiGraph, Pensacola, FL, USA), which measures three-dimensional acceleration of the body. Children wore the monitor, attached to an elastic belt, tightly fixed above the right hip for seven consecutive days including the nights. The device was removed for water-based activities, e.g. showering or swimming. PA data (raw data) were collected at a sampling frequency of 30 Hz, downloaded in three-second epochs, and aggregated to 15-s epochs. Non-wearing time, defined as a period of 20 min of consecutive zero counts, was removed. All recordings between 9 pm and 7 am were also excluded as this most likely reflected the hours spent sleeping. A minimum of three days, including one weekend day, with at least 10 h of recorded activity per day was required for inclusion in analysis, as defined a priori according to previous studies [19]. Accelerometer cut-points for moderate-to-vigorous physical activity (MVPA; min/day) and SB (min/day) were chosen based on comparability with previous literature as well as recent work comparing cut-points for various intensities in preschool children [20], which found that best classification accuracy was achieved using the Pate cut-point for MVPA (≥420 counts per 15s) [21] and the Evenson cut-point for sedentary behavior (≤25 counts per 15s) [22]. Total PA (TPA) was calculated as mean accelerometer counts per min (cpm).

### Potential correlates

Variables were selected a priori on the basis of previous research and theory guided by the socio-ecological model, and classified according to five domains [23]: (i) biological and demographic; (ii) psychological, cognitive and emotional; (iii) behavioral; (iv) social and cultural; and (v) environmental. A detailed description of all 35 potential correlates is provided in Additional file 1.

#### Biological and demographic variables

Ten biological or demographic variables were included in the analysis. Birth weight was provided in grams and entered the analysis as a continuous variable (per 100g). Chronic health condition was defined as having vs. not having a chronic health condition, e.g. asthma. Body mass index (BMI)-for-age percentiles were constructed for boys and girls separately using the WHO Child Growth Standards and categorized as normal (<85th percentile) vs. overweight and obese (≥85th percentile) [24]. Parental weight status was defined as normal-weight (BMI ≤ 25) vs. at least one overweight/obese parent (BMI > 25). Gross motor skills were assessed using the Zurich Neuromotor Assessment 3-5 (ZNA 3-5) [25, 26]. A composite z-score of individual sub-scores (walking, running, jumping, hopping) was calculated if at least three of four subtests were available. The presence of older siblings was subdivided into having older siblings vs. no older siblings in the household. Socio-economic status (SES) was assessed based on mother’s or father’s occupation (depending on who was highest) using the International Socio-Economic Index of occupational status (ISEI) [27], which assigns values between 16 and 90 to job titles with respect to education and income. Single parent family structure distinguished between single parent and dual parent households. There were no children living without any parent.

#### Psychological, cognitive and emotional variables

Seven psychological-level variables were included. Parents rated their children’s temperament using the Emotionality, Activity, and Sociability Temperament Survey (EAS) [28]. This questionnaire consists of 20 5-point rating items, five corresponding to each of the four temperament dimensions emotionality (tendency to show distress), activity (preferred levels of activity and speed of action), sociability (tendency to prefer the presence of others to being alone) and shyness (tendency to be inhibited with unfamiliar people) that together shape a child’s personality. Higher scores indicate a greater expression of the described temperamental characteristic. In our sample, mean ratings were similar to normative values of preschool children [29] and internal consistency of scales was satisfying, with Cronbach’s α ranging from α = .70 for activity to α = .71 for emotionality and α = .75 for shyness. Due to poor internal consistency for the sociability scale (α = .35), this dimension was removed from the analysis. Parenting stress was measured using the 18-item parent-report Parenting Stress Scale (PSS) [30], which assesses strain and satisfaction in parenting. Higher scores indicate more parenting stress. Norm values for parents of typically developing children averaged 37.1 (standard deviation [SD] = 8.1) [30], similar to our sample. The scale’s internal consistency for the current sample was high (α = .80). Emotional and behavioral problems were assessed using the parental version of the Strengths and Difficulties Questionnaire (SDQ) [31], which consists of five scales (emotional symptoms, conduct problems, hyperactivity/inattention problems, peer relationship problems and pro-social behavior). A ‘total difficulties’ score can be derived from the sum of scores for the emotional, conduct, hyperactivity and peer relationship problem scales. In this study, the total difficulties scale ranging from 0 to 40, with higher scores indicating more difficulties, was used. Mean values were comparable to norm values of similar age groups and reliability was between α = .49 and α = .69, hence somewhat lower than in normative data (α between .57 and .76) [32]. Cognitive functioning was assessed using the Intelligence and Development Scales – Preschool (IDS-P) [33]. A composite z-score of four individual sub-scores (visual perception, selective attention, visuo-spatial memory and figural reasoning) was calculated if at least three subtests were valid. The subtests showed good internal consistency in our sample (α = .79). As we expect correlates of self-regulation capacity to be important when it comes to the development of sustained PA, we measured motor persistence and inhibition capabilities using the NEPSY (a developmental neuropsychological assessment) Statue test [34]. Lower values on a scale from 0 to 30 indicate better suppression of responses requiring a motor response. Norm values for typically developing children averaged 21.48 (6.85) [35], which is very similar to the result observed in our sample (20.15 [9.15]).

#### Behavioral variables

Two behavioral variables were considered. Children’s parent reported hours of sleep per night during week- and weekend days was averaged over the week and frequency of play with other children (siblings, friends, neighbors) was dichotomized into playing more than once per week vs. equal to or less than once per week.

#### Social and cultural variables

Seven social-level variables were assessed via parental questionnaire. Parental sedentary time, i.e. time spent on screen-based or other sedentary activities, was calculated as the average sedentary time for both parents. Parental sports club membership was defined as at least one parent being an active (participation at least once per month) sports club member. Parental PA was categorized into neither parent vs. at least one parent meeting the PA guidelines (at least 150 min/week MVPA [36]). Parental involvement in children’s PA was dichotomized into at least one organized PA per week or at least two non-organized activities with either parent per week vs. less involvement. Typical mode of commuting to the childcare facility was divided into active vs. passive transport. Parental tobacco use was subdivided into non-smoking parents vs. at least one smoker. Parental alcohol consumption was categorized into none of the parents drinking alcohol in amounts above the upper limit of recommended intake levels based on its sex specific risk for undesirable health effects (men: 2 standard drinks per day, women: one standard drink per day [37, 38]) vs. at least one parent consuming more.

#### Environmental variables

Nine environmental-level variables were identified from parental questionnaire data; time children spend outdoors, number of portable play equipment (balls, jump ropes, etc.) and fixed play items (trampoline, swing, etc.) in the home environment, number of days children are in the childcare center, and indoor living area per person (m^2^) were included. In order to assess parental perceptions of neighborhood safety, parents were asked to indicate how much they agreed or disagreed with a series of 11 statements. These statements were related to perceptions about traffic density, road safety, crime, strangers and access to outdoor play facilities in their local area. The items were adapted from the Neighborhood Environment Walkability Scale [39] and other previously tested and validated instruments [40, 41]. A sum score with a potential range of zero to 44 was used in analysis. High scores indicate more concerns regarding neighborhood safety. Scale reliability analysis revealed a Cronbach’s α of 0.79 and construct validation by means of principal component analysis revealed a one factor solution indicating that all 11 items were meaningfully affected by one underlying dimension (neighborhood safety). Furthermore, we assessed whether a dog was kept as a pet. Season was established using the start date of accelerometer recording and categorized according to seasonal weather patterns into summer vs. autumn and spring. Based on the definition of the Organization for Economic Co-operation and Development (OECD) on urban areas [42], geographic region was dichotomized into rural (<50′000 inhabitants) vs. urban (≥50′000 inhabitants) areas.

### Statistical analysis

All analyses were performed using Stata statistical software version 13 (StataCorp. 2013, College Station, Texas, USA) or R version 3.2.3 (R Foundation for Statistical Computing, Vienna, Austria). Descriptive statistics are presented using the mean and standard deviation for continuous variables and percentages for categorical variables, unless stated otherwise.

Multilevel linear regression modeling including childcare as a random factor was used to determine correlates of TPA, MVPA and SB. Assigning childcare as a random variable in the models accounted for the potential clustering effect of the childcare with respect to the outcome of interest. Collinearity diagnostics indicated that multicollinearity was not an issue. Potential correlates were entered simultaneously in the regression model; variables for which the results indicated at least some evidence for an association with the outcome (i.e., where *p* ≤ 0.10) were subsequently included in the final model. Importantly, we also tested the robustness of this approach (see below). *P*-values obtained in the final model were used to quantify the evidence that potential correlates are associated with the outcome, i.e. to test if variables have some explanatory power. Hence, the smaller the *p*-value, the more evidence there is for a relationship between a potential correlate and the respective outcome. We denoted variables with a *p*-value ≤ 0.05 as correlates. Of those, variables with *P* ≤ 0.01 were considered strong correlates. For the final models we calculated R^2^ as a summary statistic that described the amount of variance explained. Marginal R^2^ (variance explained by fixed factors) and conditional R^2^ (variance explained by fixed and random factors) were estimated as described by Nakagawa S and Schielzeth H [43]. In addition, relative importance assessment based on variance decomposition was conducted by calculating the relative contribution of each factor to the model explained variance (R^2^). Model R^2^ decomposition was estimated using the LMG approach proposed by Lindeman RH, Merenda PF and Gold RZ [44] (implemented in R package “relaimpo” developed by Groemping U [45]). The R package “relaimpo” only supports relative importance assessment of regressors in single level but not multilevel linear models. Since in our models only a small proportion of variance was captured by the random factor (0.4–4.4%), multiple linear regression models (i.e., without the random factor) were used to compute the LMG measure of relative importance. Sensitivity analysis was conducted to test the robustness of our results, details are provided in Additional file 2.

Information on the percentage of missing data for each variable is provided in Additional file 1. With the exception of three variables, this was less than 10%. Our dataset consisted of 47% complete cases. Missing data was imputed using the MICE (Multiple Imputation Chained Equations) procedure in Stata [46]. Details on multiple imputation (MI) are provided in Additional file 3. The results presented in this work are based on imputed explanatory data. The final sample consisted of 394 (83%) children (*n* = 42 had missing outcome data, *n* = 40 had invalid outcome data).

## Results

Data were collected from 476 children and their parents. Descriptive statistics are shown in Table 1. Included participants (*n* = 394) provided an average of 5.6 (0.9) days of valid PA data with a mean wearing time of 12.8 h (0.6) per day (i.e., from 7 am to 9 pm). Mean age was 3.9 (0.7) years and 54% were boys. On average, children spent 93 (30) and 374 (48) min/day in MVPA and SB, respectively. Mean TPA was 624 (150) cpm. Participants included in the analysis did not vary from those excluded (all *p*-values above .05).

**Table 1:** Potential correlates of young children’s objectively measured physical activity and sedentary behavior (*n* = 394)

Potential correlates	Use in analysis	Mean (SD) or %
Demographic and biological variables		
Sex	Binary variable (%boys)	53.9
Age	Continuous variable (years)	3.9 (0.7)
Birth weight	Continuous variable (grams)	3297.7 (566.7)
Chronic health condition	Re-coded to dichotomous variable (%with chronic health condition)	7.6
BMI^a^	Re-coded to dichotomous variable (%overweight or obese)	24.9
Gross motor skills^a^	Continuous variable (composite z-score)	0.04 (1.0)
Siblings	Binary variable (%having older siblings)	41.8
Parental BMI	Re-coded to dichotomous variable (%at least one overweight or obese parent)	48.4
SES	Continuous variable (parental ISEI score [range 16–90; increases with higher SES])	61.5 (15.9)
Family structure	Binary variable (%single parent households)	9.8
Psychological, cognitive and emotional variables		
Self-regulation^a^	Continuous variable (NEPSY score [range 0–30; increases with better self-regulation])	20.3 (9.1)
Psychological difficulties	Continuous variable (SDQ total score [range 0–40; increases with more difficulties])	8.9 (4.5)
Emotionality temperament	Continuous variable (EAS emotionality score [range 1–5; increases with more pronounced trait])	2.8 (0.7)
Activity temperament	Continuous variable (EAS activity score [range 1–5; increases with more pronounced trait])	3.8 (0.7)
Shyness temperament	Continuous variable (EAS shyness score [range 1–5; increases with more pronounced trait])	2.3 (0.7)
Parenting stress	Continuous variable (PSS score [range 5–90; increases with more parenting stress])	37.2 (7.4)
Cognitive performance^a^	Continuous variable (composite z-score)	0.03 (0.8)
Behavioral variables		
Sleep duration	Continuous variable (hours)	10.8 (0.6)
Play frequency	Re-coded to dichotomous variable (%more than once/week)	86.4
Social and cultural variables		
Parental sedentary behavior	Continuous variable (hours)	3 (2–5)^b^
Parental sports club membership	Re-coded to dichotomous variable (%at least one parent is member)	27.9
Parental physical activity	Re-coded to dichotomous variable (%at least one parent is active)	57.9
Parental involvement in child PA	Re-coded to dichotomous variable (%at least one parent is involved)	57.9
Transport to childcare	Binary variable (%active)	38.5
Parental tobacco use	Re-coded to dichotomous variable (%at least one parent smokes)	24.7
Parental alcohol consumption	Re-coded to dichotomous variable (%at least one parent consumes large amounts)	5.0
Environmental variables		
Time outdoors	Continuous variable (hours)	2 (1.5–3.0)^b^
Fixed toys	Continuous variable (number of items [range 0–7])	1.6 (1.5)
Portable toys	Continuous variable (number of items [range 0–8])	4.4 (1.5)
Days at childcare	Continuous variable (number of days [range 0–5])	2.8 (1.2)
Living area per person	Continuous variable (m2)	30 (23.3–37.5)^b^
Neighborhood safety	Continuous variable (neighborhood safety sum score [range 0–44; increases with increasing concerns])	12.5 (6.9)
Dog	Binary variable (%dog owner)	5.8
Season	Re-coded to dichotomous variable (%summer)	24.9
Region	Binary variable (%urban)	34.3

*PA* physical activity, *BMI* body mass index, *SES* socio-economic status, *SEI* international socio-economic index

^a^Directly assessed (all other information is parent-report)

^b^Median and inter-quartile range presented for skewed distribution

Table 2 presents multivariate associations between potential correlates and TPA, MVPA and SB. Of the 13 variables associated with TPA in the full multilevel analysis (*p* ≤ 0.1), eight were identified as correlates in the final model (all *p* ≤ 0.028; Table 3). Results showed that boys were more active than girls (*p* = 0.005). Similarly, children from single parent families had a higher level of activity than those living with two parents (*p* = 0.021). Age (*p* < 0.001), gross motor skills (*p* = 0.016), time outdoors (*p* = 0.009), number of fixed play items (*p* = 0.013) and child’s activity temperament (*p* < 0.001) were positively associated with TPA. Moreover, children spent more time active in the spring and autumn months compared to summer (*p* = 0.028). For MVPA, six of a total of 11 variables associated with the outcome in the full model were identified as correlates in the final model (all *p* ≤ 0.032). Boys accumulated more MVPA than girls (*p* < 0.001). Furthermore, MVPA was positively associated with age (*p* < 0.001), birth weight (*p* = 0.032), gross motor skills (*p* = 0.001) and child’s activity temperament (*p* < 0.001). Like for TPA, children spent more time in MVPA in spring and autumn compared to summer (*p* = 0.007). When SB was analyzed as the outcome, results of the final model indicated that for six of a total of ten variables there was strong evidence for an association (all *p* ≤ 0.018). Children living in a dual-parent household (*p* = 0.002) and whose parents had no sports club membership (*p* < 0.018) spent more time sedentary. Time outdoors (*p* = 0.003) and the child’s activity temperament (*p* < 0.001) were negatively associated with SB. Concerns about neighborhood safety (*p* = 0.002) and apartment size (*p* = 0.007) were positively related to SB.

**Table 2:** Full models: associations of potential correlates with total physical activity, moderate-to-vigorous physical activity and sedentary behavior^a^

	TPA [cpm]			MVPA [min/day]			SB [min/day]		
	β	95% CI	*p*-value	β	95% CI	*p*-value	β	95% CI	*p*-value
Demographic and biological variables									
Sex	27.4	(0.1, 54.6)	0.049	11.0	(5.8, 16.2)	≤0.001	-0.3	(-9.6, 8.9)	0.946
Age	76.9	(45, 108.7)	≤0.001	15.9	(9.8, 22)	≤0.001	-8.9	(-19.9, 2.1)	0.111
Birth weight	2.3	(-0.2, 4.8)	0.076	0.6	(0.1, 1)	0.023	-0.6	(-1.5, 0.2)	0.126
Chronic health condition	-35.2	(-85.3, 15)	0.170	-5.2	(-14.8, 4.4)	0.286	8.8	(-7.8, 25.4)	0.3
BMI	20.4	(-10.4, 51.2)	0.195	4.7	(-1.2, 10.5)	0.121	-7.3	(-17.7, 3.1)	0.17
Gross motor skills	20.4	(5.2, 35.6)	0.009	4.6	(1.6, 7.6)	0.003	-3.3	(-8.6, 2.1)	0.229
Siblings	-38.9	(-67.7, -10)	0.008	-6.3	(-11.8, -0.8)	0.025	8.7	(-1, 18.4)	0.079
Parental BMI	-4.4	(-33.2, 24.4)	0.763	0.0	(-5.4, 5.5)	0.994	-5.2	(-15, 4.5)	0.293
SES	-0.2	(-1.2, 0.9)	0.758	-0.1	(-0.3, 0.1)	0.511	-0.1	(-0.4, 0.3)	0.698
Family structure	64.3	(15.5, 113.1)	0.010	9.5	(0.2, 18.7)	0.045	-27.5	(-43.6, -11.4)	0.001
Psychological, cognitive and emotional variables									
Self-regulation	-0.4	(-2.2, 1.5)	0.704	0.0	(-0.4, 0.3)	0.959	0.5	(-0.1, 1.2)	0.099
Psychological difficulties	1.4	(-2.5, 5.3)	0.484	0.4	(-0.4, 1.1)	0.346	-0.3	(-1.7, 1)	0.619
Emotionality temperament	-18.4	(-41.7, 4.9)	0.122	-3.6	(-8.1, 0.9)	0.114	4.5	(-3.2, 12.3)	0.252
Activity temperament	39.6	(18.2, 60.9)	≤0.001	6.9	(2.8, 10.9)	0.001	-15.6	(-22.9, -8.4)	≤0.001
Shyness temperament	-14.0	(-37.1, 9.2)	0.236	-0.9	(-5.3, 3.5)	0.691	2.1	(-5.6, 9.7)	0.593
Parenting stress	1.7	(-0.4, 3.8)	0.114	0.3	(-0.1, 0.7)	0.167	-0.1	(-0.8, 0.6)	0.822
Cognitive performance	-18.0	(-46.8, 10.8)	0.222	-2.3	(-7.8, 3.1)	0.401	4.5	(-5.6, 14.5)	0.385
Behavioral variables									
Sleep duration	-7.2	(-28.5, 14.2)	0.510	-1.0	(-5.1, 3)	0.614	-1.3	(-8.5, 5.9)	0.714
Play frequency	3.7	(-46.6, 54.1)	0.884	0.6	(-9.1, 10.2)	0.911	-0.8	(-17.6, 16)	0.923
Social and cultural variables									
Parental sedentary behavior	0.5	(-4.1, 5.2)	0.829	0.2	(-0.7, 1.1)	0.673	0.2	(-1.4, 1.8)	0.808
Parental sports club membership	34.4	(0.9, 67.9)	0.044	4.7	(-1.7, 11.1)	0.151	-12.7	(-24.2, -1.3)	0.029
Parental physical activity	18.2	(-11.6, 48.1)	0.231	2.9	(-2.8, 8.6)	0.314	-9.2	(-19.3, 0.8)	0.072
Parental Involvement in child PA	3.0	(-28.9, 34.9)	0.853	-2.1	(-8.2, 3.9)	0.485	-5.3	(-15.6, 4.9)	0.308
Transport to childcare	0.6	(-29.2, 30.4)	0.969	2.1	(-3.6, 7.8)	0.475	-3.2	(-13.1, 6.8)	0.531
Parental tobacco use	11.4	(-20.4, 43.2)	0.483	3.5	(-2.5, 9.6)	0.254	0.1	(-10.7, 10.9)	0.987
Parental alcohol consumption	-62.0	(-122.9, -1.1)	0.046	-11.9	(-23.6, -0.1)	0.047	22.6	(2.3, 42.9)	0.029
Environmental variables									
Time outdoors	11.8	(2.1, 21.5)	0.017	1.6	(-0.2, 3.5)	0.081	-4.4	(-7.7, -1.1)	0.008
Fixed toys	11.5	(1.3, 21.7)	0.028	1.5	(-0.4, 3.4)	0.129	-2.4	(-5.7, 1)	0.17
Portable toys	6.4	(-3.8, 16.6)	0.216	1.3	(-0.7, 3.2)	0.201	-0.6	(-4, 2.9)	0.75
Days at childcare	-5.0	(-22.1, 12)	0.565	-1.2	(-4.8, 2.4)	0.505	1.2	(-5.4, 7.8)	0.72
Living area per person	-1.3	(-2.9, 0.4)	0.137	-0.2	(-0.5, 0.2)	0.338	0.7	(0.1, 1.2)	0.019
Neighborhood safety	-2.3	(-4.4, -0.1)	0.043	-0.4	(-0.9, 0)	0.040	1.0	(0.3, 1.8)	0.006
Dog	-31.3	(-89.6, 26.9)	0.291	-6.8	(-17.8, 4.2)	0.227	15.2	(-3.9, 34.4)	0.119
Season	42.6	(11.7, 73.4)	0.007	9.1	(3.2, 15)	0.002	-7.1	(-17.5, 3.4)	0.187
Region	2.9	(-28.2, 33.9)	0.857	2.0	(-3.9, 7.9)	0.506	-5.2	(-15.5, 5.1)	0.325

*PA* physical activity, *β* β-coefficient, *CI* confidence interval

^a^Multilevel linear model with childcare as a random factor including all potential correlates (*n* = 394)

**Table 3:** Final models: associations of correlates with with total physical activity, moderate-to-vigorous physical activity and sedentary behavior^a^

	TPA [cpm]			MVPA [min/day]			SB [min/day]		
	β	95% CI	*p*-value	β	95% CI	*p*-value	β	95% CI	*p*-value
Demographic and biological variables									
Sex	36.8	(10.8, 62.8)	0.005	11.8	(6.9, 16.8)	≤0.001	-	-	-
Age	61.1	(42.2, 79.9)	≤0.001	14.4	(10.8, 18.1)	≤0.001	-	-	-
Birth weight	2.0	(-0.4, 4.5)	0.109	0.5	(0.0, 1.0)	0.032	-	-	-
Gross motor skills	16.9	(3.2, 30.5)	0.016	4.6	(1.9, 7.2)	0.001	-	-	-
Siblings	-25.5	(-52.5, 1.5)	0.064	-3.0	(-8.2, 2.1)	0.248	7.0	(-2.26, 16.34)	0.138
Family structure	55.4	(8.3, 102.5)	0.021	7.5	(-1.3, 16.3)	0.094	-24.8	(-40.4, -9.3)	0.002
Psychological, cognitive and emotional variables									
Self-regulation	-	-	-	-	-	-	0.4	(-0.1, 0.9)	0.119
Activity temperament	43.6	(24.7, 62.5)	≤0.001	7.7	(4.1, 11.3)	≤0.001	-17.3	(-23.7, -10.9)	≤0.001
Social and cultural variables									
Parental sports club membership	26.8	(-3.9, 57.6)	0.087	-	-	-	-13.2	(-24.2, -2.3)	0.018
Parental physical activity	-	-	-	-	-	-	-8.1	(-17.9, 1.6)	0.103
Parental alcohol consumption	-51.2	(-110.8, 8.3)	0.092	-9.0	(-20.4, 2.3)	0.118	18.3	(-1.6, 38.2)	0.071
Environmental variables									
Time outdoors	12.5	(3.2, 21.9)	0.009	1.5	(-0.3, 3.2)	0.105	-4.7	(-7.8, -1.6)	0.003
Fixed toys	12.3	(2.6, 22.0)	0.013	-	-	-	-	-	-
Living area per person	-	-	-	-	-	-	0.7	(0.2, 1.2)	0.007
Neighborhood safety	-1.9	(-3.9, 0.1)	0.068	-0.3	(-0.7, 0.0)	0.074	1.1	(0.4, 1.8)	0.002
Season	34.2	(3.8, 64.5)	0.028	8.1	(2.2, 13.9)	0.007	-	-	-

*β* β-coefficient, *CI* confidence interval

^a^Multilevel linear model with childcare as a random factor including all variables with *p*-value ≤ 0.1 in the full model (*n* = 394)

**Fig. 1:**
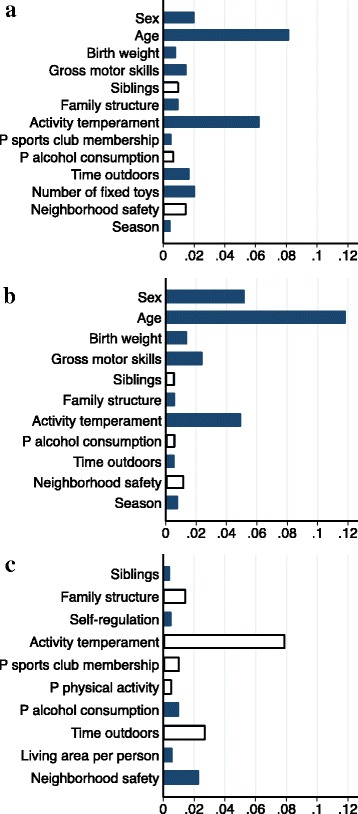
Relative importance of correlates to each of the three outcomes TPA (**a**), MVPA (**b**) and SB (**c**). Because estimators of relative importance are based on variance decomposition, adding up estimators for each outcome corresponds to the proportion of variance explained by the fixed factors, i.e. 28% (**a**), 30% (**b**) and 17% (**c**). Shaded bars indicate positive associations, open bars indicate inverse associations

The fixed effects in the final models explained 28%, 30% and 17% of the variance (marginal R^2^) in TPA, MVPA and SB, respectively. The proportion of variance explained in TPA, MVPA and SB including all fixed effects plus the random effect (conditional R^2^) was 28%, 32% and 22%, respectively, indicating that the random factor did not capture a lot of additional variance. Figure 1 shows the relative importance of variables included in the final models, i.e. the proportion of variance in the response variable explained by each explanatory variable. Note that these values add up to the variance explained by the fixed factors.

**Fig. 2:**
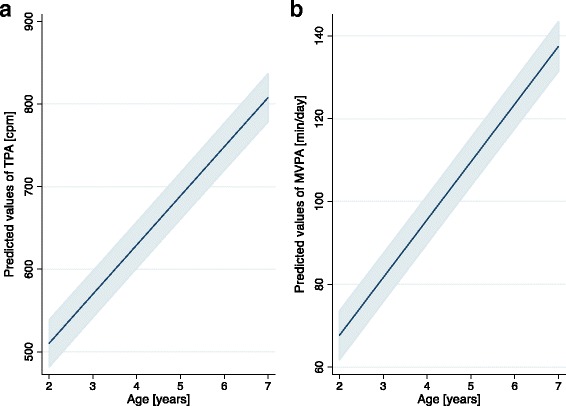
Marginal predicted TPA and MVPA by age with 95% confidence intervals. Based on final models of Table 3 for TPA (**a**) and MVPA (**b**), holding all other variables constant

Age was found to be the most important correlate of both, TPA and MVPA; older children were more active than younger ones. To illustrate the relationship of age with PA, plots of marginal predictions of PA progression by age, as predicted from the final models for TPA and MVPA, are shown in Fig. 2. Other factors of high relative importance to PA were sex (boys more active than girls) and activity temperament (increased PA with more pronounced activity temperament). For TPA, number of fixed toys and time outdoors, for MVPA, gross motor skills and birth weight, were positively associated and played a major role. Factors that were important to SB include activity temperament, time outdoors, neighborhood safety, family situation and parental sports club membership, all of which were inversely related to SB except for neighborhood safety. Including age and sex into the final model for SB neither noticeably changed effect estimates nor variance explained. Sensitivity analyses revealed that the results from our final models were robust, i.e. the variable selection procedure used for identification of correlates was adequate (see also Additional file 2).

## Discussion

Guided by the behavioral epidemiology framework and the socio-ecological model, this study aimed at identifying correlates of PA and SB in preschool children by investigating known, under-researched and novel associations between biological/demographical, psychological/cognitive, behavioral, social/cultural and environmental factors and objectively measured TPA, MVPA and SB. For 12 of the 35 potential correlates there was evidence for an association (*p* ≤ 0.05) with at least one outcome variable; the direction of association was positive for the PA correlates and, with the exception of neighborhood safety and living area, negative for the SB correlates. Four correlates can be classified as at least to some extent modifiable: gross motor skills, parental sports club membership, time outdoors and fixed home play equipment. These factors could be targeted in interventions aiming at promoting healthy physical activity behaviors [47]. Of the correlates identified, three were associated with both, a PA outcome and SB, six correlates were found to be associated with PA, four of which were related to both TPA and MVPA, and another three correlates were related to SB only. This suggests that some common factors may influence PA and SB in young children. However, most factors were associated with either PA or SB, which illustrates that correlates of these behaviors/constructs may differ and should ideally be considered separately [48]. Besides activity temperament, demographic and biological variables had the highest relative importance to PA, which has previously been found [49], whereas variables from the environmental domain were most influential for SB.

Our study identified a number of previously unreported correlates of preschoolers’ PA and SB, including single-parent family structure, child’s activity temperament, number of fixed play equipment and home living area per person. Relatively little work has been conducted on the putative influences on young children’s SB. Hence, reviews concluded that there was insufficient evidence to draw conclusions regarding correlates of SB in young children [17, 50]. On the other hand, consistent correlates of preschool children’s PA identified in reviews exist [14–16]. These include gender (male preschoolers are more active than female preschoolers), parental PA (parent’s PA is positively associated with their child’s PA), parents’ participation in children’s PA (children of parents who actively engage in PA with their children are more active than children of parents who do not participate), time outdoors (outdoor play time is positively associated with PA) and gross motor skills (MVPA is positively associated with gross motor skills). In line with these findings, we identified gender, time outdoors and gross motor skills, but not parental PA and parents’ participation in PA, as positive correlates of PA.

Relative importance analysis revealed that age was the most influential correlate of TPA and MVPA. While in late childhood and adolescence the decline in PA with age is a common finding, this trend is less evident in younger children [23]. Several reviews have reported no age effect in the preschool population [14, 16, 51]. In this study, we found that older preschool-aged children were more physically active than younger ones; both TPA and MVPA were positively associated with age and increased by an average of 10% and 16%, respectively, per year (see Fig. 2). The increase in PA with age found in our study could not be attributed to differences in wear-time, as a minimum of ten hours of recorded activity per day was required and no significant differences in hours/day of recorded activity were found between younger and older children. In fact, this pattern has been replicated using energy expenditure [52] and pedometry [53]. This supports the hypothesis that rather than being the result of other moderating factors or issues relating to standardization of PA data collection and processing, an actual difference in activity behavior by age seems plausible. Different contexts, policies and practices, may have a substantial influence in this regard [54].

A novel finding of the current study is the association of family structure with activity patterns, which was shown to be particularly relevant for SB; single parent status was positively associated with children’s TPA and negatively related to SB. Studies on the influence of single parent family status on children’s activity, which include preschool children, have previously been published. However, none have reported results specific to the preschool population [16]. Support for a positive association was found in a study with school-aged children conducted in southern California [55]. Furthermore, a review by Sallis et al. [23] concluded that single parent status was indeterminately related to children’s (aged 3–12 years) PA, whereas a meta-analysis published in 2015 [56] found no differences in objectively measured PA between children (aged 6–18 years) living in single parent families compared to those living with two parents. One possible explanation for our finding is based on the need for constant supervision of the young child by the single caregiver, which may lead to higher levels of activity because the caregiver always needs to take the child along when getting things done (e.g., grocery shopping). In this context it is worth mentioning that childcare attendance did not differ between children from single parent vs. dual parent families, i.e. childcare attendance was not relevant to the explanation of the observed difference in activity. The association of PA and SB with family structure could also arise from the effect of modeling and the potential role of compensation by the existing parent [57]. It has been shown that having only one role model is better than two negative, i.e. physically inactive role models [58]. This may indicate the power of negative role modeling, or it may imply that the remaining parent tries to compensate for the lack of role models. While a difference of 55 cpm for TPA appears relatively small, an increase in TPA of 50 cpm was shown to be associated with a 2 mm Hg reduction in current and future blood pressure of children aged five to seven years [59]. Although the clinical relevance of this difference in children is not entirely clear, a 2 mm Hg reduction in blood pressure in adults is associated with a 6% reduction in coronary heart disease and a 15% reduction in the risk of stroke and transient ischemic attacks [60].

The child’s activity temperament, as assessed by the EAS temperament survey to determine the child’s preferred levels of activity and speed of action, was strongly (positively for PA, inversely for SB) related to all outcome variables. This suggests that the activity domain is relevant to the prediction of objectively measured TPA, MVPA and SB among preschool children. Our findings indicate that the child’s temperament is associated with his or her choice to engage in PA and sedentary pursuits already very early in life. If this pattern is confirmed in further studies, researchers will be better positioned to find strategies that are adapted to children of different temperaments. A previous cross-sectional study found no relationship between preschoolers’ temperament assessed using the Child Temperament Questionnaire (CTQ) and objectively measured PA and sedentary time [61]. It should be noted that high scores on the temperament dimension of activity neither imply poor attention, nor serve as diagnostic criteria for attention deficit hyperactivity disorder (ADHD) or as a precursor of symptoms of this disorder. Our findings rather provide support for the validity of the survey’s activity domain to accurately depict young children’s activity behavior by parent report.

In contrast to expectations, parental behaviors such as parental PA, parental support (i.e., involvement in child PA) and role modeling (i.e., parental PA and SB) were not found to be associated with children’s PA and SB. Only parental sports club membership was identified as a correlate of SB. However, while some factors have elicited substantial changes in PA, the clinical relevance of a 13 min difference in SB, as seen for sports club participation, requires further evaluation. The same applies to other factors, such as neighborhood safety, where the influence on SB seems clinically negligible despite strong evidence for an association and considerable relative importance.

Key strengths of the present study include the integration of an extended set of potential correlates of PA and SB across five domains of the socio-ecological model, the study’s multi-method approach including direct measurements as well as parent-report information, the use of objective, reliable, and validated measures of PA and SB, the short epoch lengths of activity recording used [62], and the relatively large and representative study sample. Limitations include the cross-sectional design limiting conclusions regarding causation and the reliance on parent-reported data for some exposure variables. In addition, accelerometers tend to underestimate PA due to the inability to accurately detect certain activities (e.g., water-based activities) and there is a lack of agreement on some of the methodological aspects of PA data collection and processing, such as assessment techniques, accelerometer wear-time and accelerometer cut points [63]. Analysis of variance explained in the outcome revealed that the set of exposures studied in this work performed slightly better in explaining variance in PA rather than SB. Although previous investigations have reported similar proportions of variance explained [49], the results indicate that additional factors such as genetic traits or aspects related to different contexts, policies and practices (e.g. the childcare environment) [54] as well as other objectively assessed factors may have a substantial influence on preschool children’s activity levels.

## Conclusions

Twelve correlates of preschool children’s PA and SB across various socio-ecological domains have been identified, four of which are modifiable. Our findings provide evidence for the multidimensional nature of correlates of young children’s activity behavior and give an insight into the relative importance of different influences. Personal factors were found to have the greatest influence on PA, whereas environmental factors had the greatest influence on SB. Further longitudinal or intervention studies may reveal causality of our findings as well as evidence of the single or combined effect of known or novel correlates of young children’s PA behaviors.

## Additional files

Additional file 1: Potential correlates. (PDF 139 kb)
Additional file 2: Sensitivity analysis. (PDF 194 kb)
Additional file 3: Multiple imputation procedure. (PDF 70 kb)

## Abbreviations

BMI: Body mass index
cpm: Counts per minute
EAS: Emotionality, activity, and sociability temperament survey
ISEI: International socio-economic index
MI: Multiple imputation
min: Minute
MVPA: Moderate-to-vigorous physical activity
OECD: The organization for economic co-operation and development
PA: Physical activity
SB: Sedentary behavior
SD: Standard deviation
SES: Socio-economic status
TPA: Total physical activity
WHO: World Health Organization
