# Supplementary material for: Correlates of preschool children’s objectively measured physical activity and sedentary behavior: a cross-sectional analysis of the SPLASHY study
Source: Int J Behav Nutr Phys Act. 2017 Jan 5;14:1. doi: 10.1186/s12966-016-0456-9 (PMC5216527; doi:10.1186/s12966-016-0456-9)
Supplement: Additional file 2: — Sensitivity analysis. (PDF 194 kb) [file 12966_2016_456_MOESM2_ESM.pdf]

Additional file 2: Sensitivity analysis

Sensitivity analysis was conducted to test the robustness of our results. Since variable selection in regression is challenging, we aimed at verifying that the correlates in the final model form the best subset among all variables. The original approach selects variables based on highest ranks according to p-values and sets the cut-off at $P \leq 0.1$ for the inclusion of variables from the full model to the final model. In sensitivity analysis, a p-value of 0.25 was used as a threshold to set up the model. Based on this model, relative importance based on model R^2 decomposition was calculated [1, 2], i.e. for each model the sum of relative importance values corresponds to the model variance explained. Because the number of variables in the full model exceeds the vector size limit, the subset of variables with $p \leq 0.25$ (from the full multilevel model) was used. **Figure 1** provides a summary of the results of the two approaches for TPA (A), MVPA (B) and SB (C). Indicated in the right margin are the p-values of variables in the final models, blue bars show the corresponding relative importance. Red bars indicate relative importance from sensitivity analysis. The figure clearly suggests that the results obtained from the original approach are in line with the results from the procedure used for sensitivity analysis. This confirms the robustness of our results.

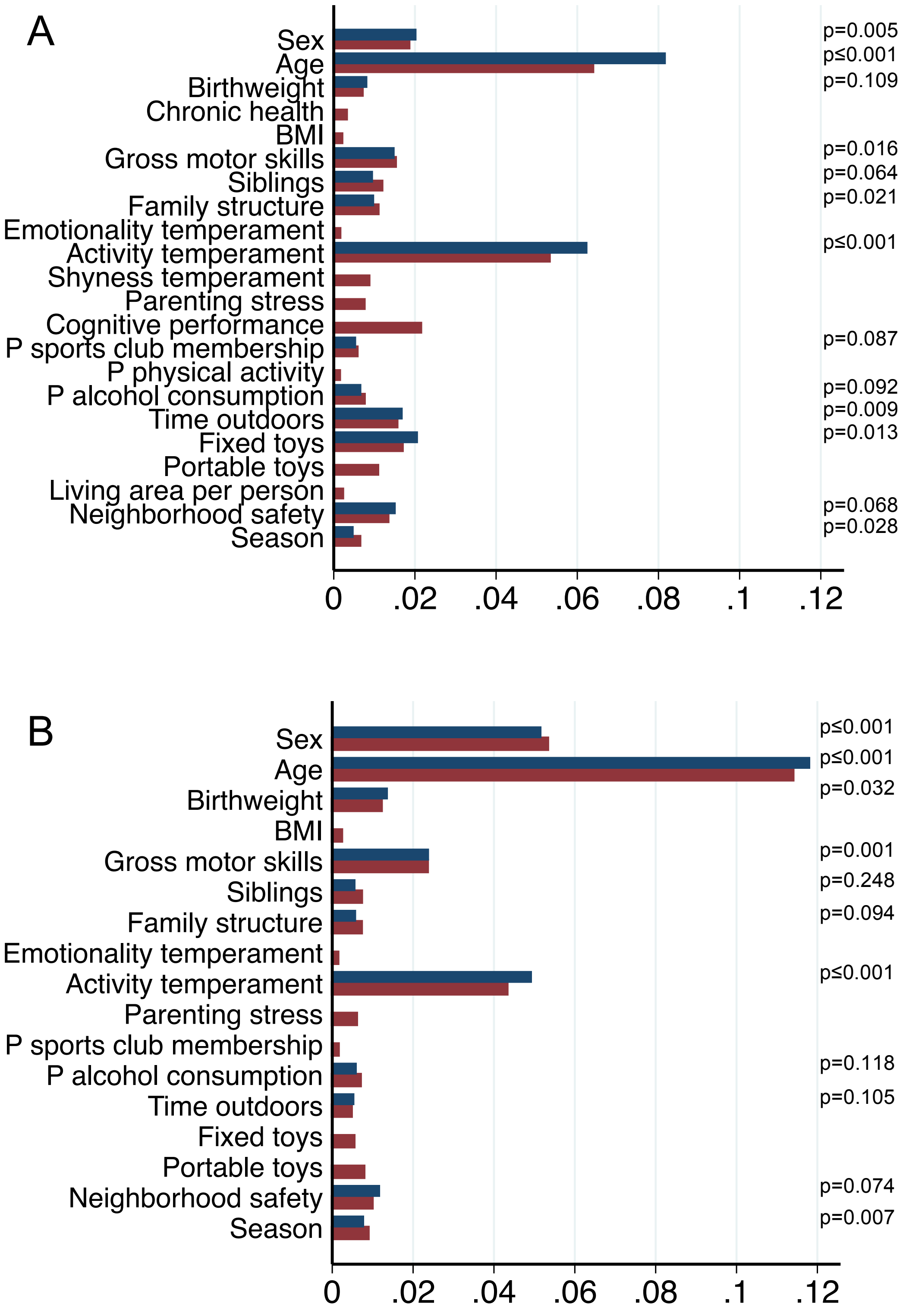
A
Sex
Age
Birthweight
Chronic health
BMI
Gross motor skills
Siblings
Family structure
Emotionality temperament
Activity temperament
Shyness temperament
Parenting stress
Cognitive performance
P sports club membership
P physical activity
P alcohol consumption
Time outdoors
Fixed toys
Portable toys
Living area per person
Neighborhood safety
Season
p=0.005
p≤0.001
p=0.109
p=0.016
p=0.064
p=0.021
p≤0.001
p=0.087
p=0.092
p=0.009
p=0.013
p=0.068
p=0.028
0
.02
.04
.06
.08
.1
.12
B
Sex
Age
Birthweight
BMI
Gross motor skills
Siblings
Family structure
Emotionality temperament
Activity temperament
Parenting stress
P sports club membership
P alcohol consumption
Time outdoors
Fixed toys
Portable toys
Neighborhood safety
Season
p≤0.001
p≤0.001
p=0.032
p=0.001
p=0.248
p=0.094
p≤0.001
p=0.118
p=0.105
p=0.074
p=0.007
0
.02
.04
.06
.08
.1
.12

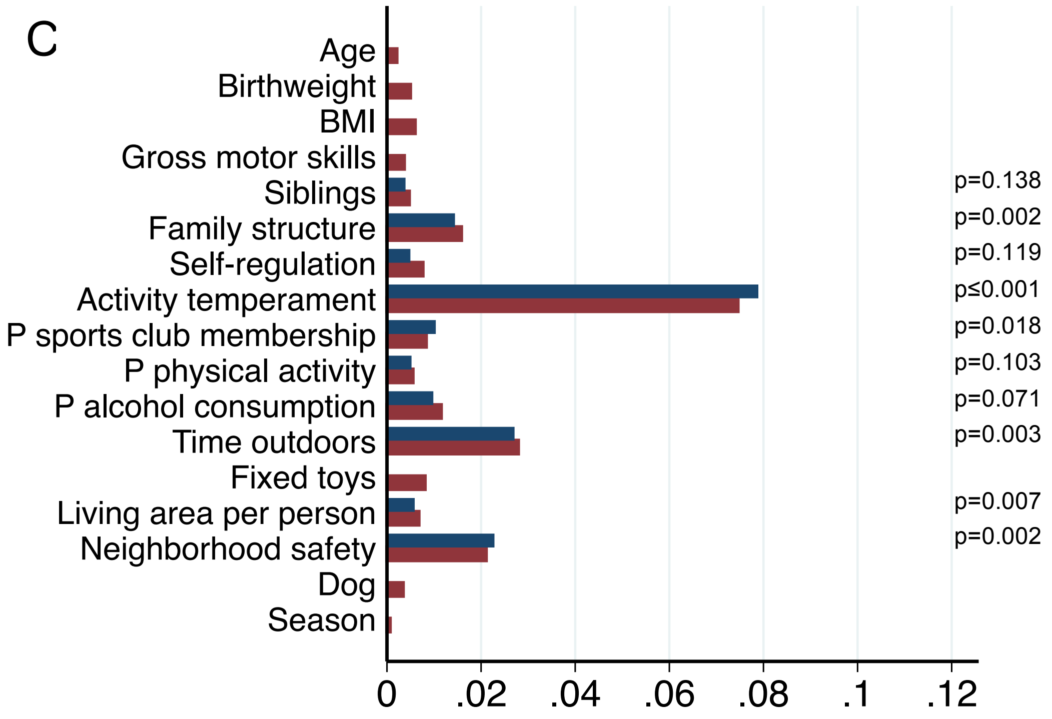

Figure 1. Comparison of results from the original approach to those obtained in sensitivity analysis for TPA (A), MVPA (B) and SB (C). Indicated in the right margin are the p-values of variables in the final models, blue bars show the corresponding relative importance. Red bars indicate relative importance from sensitivity analysis.

References

1. Groemping U: **Relative Importance for Linear Regression in R: The Package relaimpo.** *Journal of Statistical Software; Vol 1, Issue 1 (2007)* 2006.
2. Lindeman RH, Merenda PF, Gold RZ: *Introduction to Bivariate and Multivariate Analysis.* Glenview, IL: Scott Foresman; 1980.
