# Supplementary material for: Correlates of preschool children’s objectively measured physical activity and sedentary behavior: a cross-sectional analysis of the SPLASHY study
Source: Int J Behav Nutr Phys Act. 2017 Jan 5;14:1. doi: 10.1186/s12966-016-0456-9 (PMC5216527; doi:10.1186/s12966-016-0456-9)
Supplement: Additional file 3: — Multiple imputation procedure. (PDF 70 kb) [file 12966_2016_456_MOESM3_ESM.pdf]

**Additional file 3: Multiple imputation procedure**

Missing data was imputed using the MICE (Multiple Imputation Chained Equations) procedure in Stata [1]. Under a missing-at-random assumption, multiple imputation (MI) generally allows for less biased and more powerful estimates than a complete case analysis [2]. MI was performed 40 times. The imputation model contained all variables included in the analysis model to avoid bias in analyses [3]. There was no evidence of statistical heterogeneity between observed and imputed values. Analyses restricted to complete cases revealed no substantial differences from those based on MI. The final sample consisted of 394 (84%) children; n=42 with imputed outcome variables have been excluded from the analysis because including them only adds noise to the estimates [3], n=40 had invalid outcome data according to validity criteria defined a priori (see section “Outcome variables”).

**References**

1. Royston P, White IR: **Multiple Imputation by Chained Equations (MICE): Implementation in Stata.** *Journal of Statistical Software* 2011, **45:**1-20.
2. White IR, Carlin JB: **Bias and efficiency of multiple imputation compared with complete-case analysis for missing covariate values.** *Statistics in Medicine* 2010, **29:**2920-2931.
3. White IR, Royston P, Wood AM: **Multiple imputation using chained equations: Issues and guidance for practice.** *Stat Med* 2011, **30:**377-399.
